# Chromatin dynamics during plant sexual reproduction

**DOI:** 10.3389/fpls.2014.00354

**Published:** 2014-07-24

**Authors:** Wenjing She, Célia Baroux

**Affiliations:** Institute of Plant Biology – Zürich-Basel Plant Science Center, University of ZürichZürich, Switzerland

**Keywords:** chromatin dynamics, epigenetic reprogramming, histone modification, DNA methylation, nucleosome remodeling, small RNA, histone variants, plant sexual reproduction

## Abstract

Plants have the remarkable ability to establish new cell fates throughout their life cycle, in contrast to most animals that define all cell lineages during embryogenesis. This ability is exemplified during sexual reproduction in flowering plants where novel cell types are generated in floral tissues of the adult plant during sporogenesis, gametogenesis, and embryogenesis. While the molecular and genetic basis of cell specification during sexual reproduction is being studied for a long time, recent works disclosed an unsuspected role of global chromatin organization and its dynamics. In this review, we describe the events of chromatin dynamics during the different phases of sexual reproduction and discuss their possible significance particularly in cell fate establishment.

## INTRODUCTION

**FIGURE 1:**
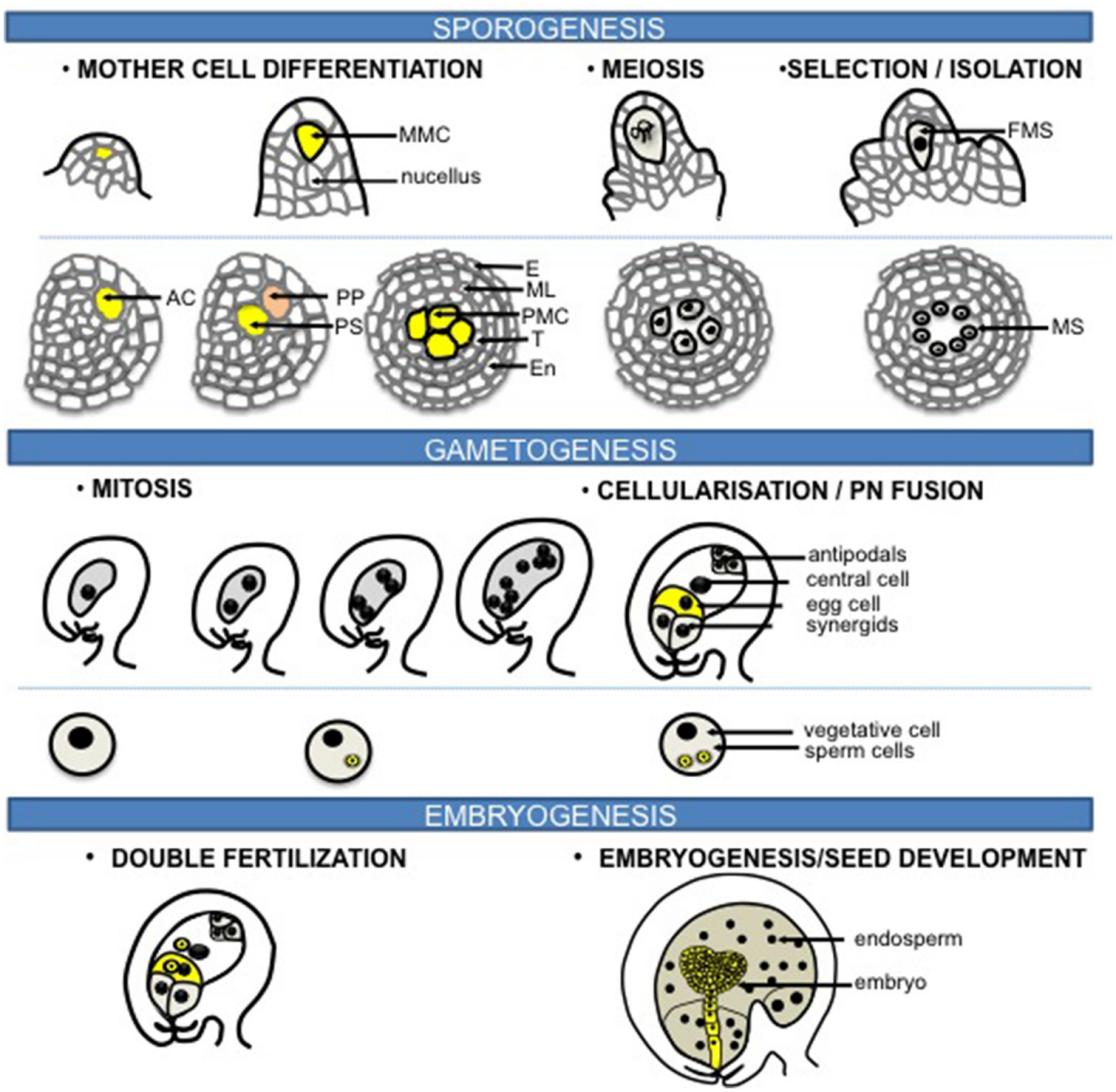
**Sexual reproduction in flowering plants.** The process of sexual reproduction begins with sporogenesis where spore mother cells (SMCs) differentiate in the floral organs of adult plants. The female SMC, also called megaspore mother cell (MMC) differentiates from a subepidermal nucellar cell within the ovule primordium, the MMC then undergoes meiosis to produce four haploid spores while only one survives to form the functional megaspore (FM). In the stamen primordium, one subepidermal cell enlarges to from the archesporial cell (AC). The archesporial cell then divides to form one primary sporogenous cell (PS) on the inner side and one primary parietal cell (PP) toward the outside. The primary parietal cell divides periclinally and anticlinally to generate the anther wall that is composed of epidermis (E), endothecium (En), the middle layer (ML), and the tapetum (T), while the primary sporogenous cell divides to give rise to the male SMCs, also called the pollen mother cells (PMCs). Each PMC then undergoes meiosis to form four haploid microspores (MS). During gametogenesis, the FM undergoes three rounds of mitosis and cellularization to generate the female gametophyte that harbors two gametes: the egg cell and the central cell, accompanied with three antipodals and two synergids. While for the male side, each microspore undergoes an asymmetric division to give rise to a larger vegetative cell and a smaller generative cell within the bicellular pollen grain. The generative cell divides further to produce the gametes: two sperm cells. During double fertilization, the egg cell is fertilized by one sperm to form the zygote that will give rise to the embryo, while the central cell fuses with the other sperm to generate the triploid endosperm. Original drawings were made after microscopy pictures (female sporogenesis) or inspired from Zhang et al. (2011) (male sporogenesis).

Flowering plants have a life cycle alternating between a dominant, diploid sporophytic phase and a short haploid gametophytic phase. Sexual reproduction can be divided into three phases: sporogenesis, gametogenesis, embryo- and endosperm-genesis (**Figure 1**). Unlike animals, plants do not set aside a germline lineage during embryogenesis. Instead, the reproductive lineage is established late in development. Cells that will share a meiotic fate and hence initiate a “reproductive lineage” differentiate from and within a somatic tissue in dedicated floral organs of adult plants. Sporogenesis is initiated by the differentiation of spore mother cells (SMCs) that engage somatic cells into a meiotic fate entailing the development of haploid, multicellular gametophytes. The female SMC, also called megaspore mother cell (MMC) differentiates in a subepidermal position in an ovule primordium – composed of the L1-outer layer of cells and the nucellus –; the male SMC differentiates from a mitotic division of the archesporial cell within the sporangium of the anther locule (**Figure 1**, and see section Chromatin Dynamics During Sporogenesis). Gametogenesis is the process by which the gametes are formed within the gametophytes. The male and female gametophytes develop from one haploid spore through a limited number of mitosis and cellularization events that will give rise to highly distinct cell types. A vast majority of flowering plants share the seven-celled type of female gametophyte comprising two gametes – the egg cell and the central cell – and five accessory cells – two synergids and three antipodals. All cells are haploid except for the central cell that inherits two polar nuclei, which following fusion generate a di-haploid maternal genome in the central cell. In contrast, the mature male gametophyte contained in the pollen grain is highly reduced and is composed of one vegetative – accessory – cell and two gametes, the sperm cells (Maheshwari, 1950; **Figure 1**). During double fertilization, the egg cell fuses with one sperm to give rise to the diploid zygote, while the central cell is fertilized by the second sperm cell – from the same pollen – to produce the triploid endosperm (**Figure 1**). Strikingly, although genetically identical the two fertilization products share distinct developmental fates. The totipotent zygote engages into embryogenesis that establishes the basic body plan and the symmetries (axial and radial) of the future seedling; in contrast, the primary endosperm cell engages in a syncytial phase of proliferation, before cellularization, to form an extra-embryonic, nurturing tissue (Maheshwari, 1950).

Genetic analyses uncovered several molecular factors responsible for cell fate establishment during plant sporogenesis, gametogenesis, and embryogenesis that shed light on the principles of cell specification during these developmental processes, underlying both commonalities and differences with cell specification in the animal reproductive lineage. Several putative intercellular signaling components, non-cell autonomous epigenetic regulators, environmental cues fueled the idea that SMC specification results from a cross-talk within the cells of the founder niche involving molecular, epigenetic, and physiological cues (reviewed in Feng et al., 2013). In contrast, cell specification in the multicellular female gametophytes involves position cues, nuclear migration, and spatially controlled cellularization (reviewed in Drews and Koltunow, 2011; Sprunck and Gross-Hardt, 2011; Rabiger and Drews, 2013). In the male gametophyte, germ cell fate commitment is contributed by factors that influence asymmetric division, cytokinesis and cell cycle (Berger and Twell, 2011), in addition to a cross-talk between the gametophytes and its surrounding tissue (reviewed in Feng et al., 2013). During embryogenesis, cell fate establishment is contributed by embryo-specific transcription factors, signaling components, and local auxin gradients overriding geometric rules of morphogenesis (reviewed in Wendrich and Weijers, 2013; Yoshida et al., 2014), but also by peptides acting non-cell autonomously (Costa et al., 2014). While still incomplete, our understanding of cell specification during plant reproduction at the genetic, molecular, physiological, and biomechanical levels improved tremendously. Yet, the current models omit a deeper level of possible control over those processes conveyed by nuclear organization. This level is, yet, still difficult to comprehend as it remains at its infancy of formulation, particularly in the field of research in plants. Nuclear organization is a collective term that describes structural and functional arrangements of the chromatin and chromatin-associated structures or factors, at the global, nuclear level, that influences *in fine* genome expression, hence the cellular phenotype; we will focus the discussion in this review onto chromatin dynamics events underlying, and perhaps partly driving, cellular fate transitions during sporogenesis, gametogenesis, and embryogenesis.

In multicellular organisms, cellular identities are the output of distinctive transcriptional programs, which in turn reflect differential, epigenetic instructions encoded beyond the genetic sequence information. Genome expression is modulated in part by the chromatin structure which influences the accessibility and processivity of the transcription machinery (Jenuwein and Allis, 2001). Two manifestations of chromatin can be discerned: an open, transcriptionally permissive state, and a compact, transcriptionally repressive state. Large-scale manifestations of these two chromatin states are microscopically visible in the nucleus as euchromatin and heterochromatin, respectively. At the cytogenetic level, while heterochromatin is typically enriched in DNA methylation, H3K9me1/2, H3K27me1/2, and H4K20me1, euchromatin is characterized by bivalent instructions such as those associated with a transcriptionally repressive (e.g., H3K27me3), and transcriptionally permissive state (H3K4me2/3, H3K9me3, H3K36me3, H3K56Ac, and H2Bub; Fransz et al., 2006; Roudier et al., 2011). The distribution of histone and DNA methylation marks along the genome is described by chromatin profiling methods. These approaches revealed that, in somatic tissues, their differential combination within promoter or core gene regions indexes distinct chromatin states (Roudier et al., 2011). Moreover, DNA methylation is observed in three sequence contexts that are enriched with gene bodies (CG) or repeat regions (CHG, CHH, respectively, Chan et al., 2005). Histone modifications and DNA methylation are set and maintained by a cohort of enzymes, with complex interplay between themselves and chromatin remodelers but also with small RNAs acting as *trans* signals that reinforce heterochromatic states (reviewed in Tariq and Paszkowski, 2004).

Heterochromatin domains cytologically defined as chromocenters contain rDNA, transposons, centromeric, and pericentromeric repeats, while euchromatin domains are composed of the distal chromosome arms deployed as rosette loops around chromocenters at interphase (Fransz and de Jong, 2002). Although chromosome territories are arranged in randomly in somatic *Arabidopsis* cells (Pecinka et al., 2004), the regular spacing of chromocenters indicates spatial constraints among chromosomes (Andrey et al., 2010). Chromosome capture-based interaction mapping revealed multiple sites that may associate with regions sharing similar chromatin states among distal chromosomal regions (Grob et al., 2013). Whether those interactions causally influence gene expression remains to be determined.

Chromatin dynamics are referred to as the processes that modify the organization of eu- and hetero-chromatin domains in the nucleus, the distribution of genomic sequences within these domains, the arrangement of chromosome territories, and the distribution of functional chromatin proteins and histone modifications. How chromatin dynamics underlie genome expression, or vice versa, particularly during cellular differentiation remains largely unknown. The aim of this review is to discuss the emerging concept that chromatin dynamics contributes to the establishment of new cell fates during sexual reproduction, and probably to the resetting of the epigenome to a ground-state toward pluripotency in the gametophyte and totipotency in the zygote.

## CHROMATIN DYNAMICS DURING SPOROGENESIS

Sporogenesis initiates with the differentiation of SMCs. The female SMC, also called MMC corresponds in *Arabidopsis* to a single sub-epidermal cell at the distal end of each ovule primordium (**Figure 1**, Maheshwari, 1950). In some species, the archesporial cell undergoes division to give rise to several MMCs (Maheshwari, 1950). The MMC undergoes meiosis to produce four haploid spores, while only one survives to form the functional megaspore (**Figure 1**). Male SMCs, also called pollen mother cells (PMCs), or microspore mother cells, differentiate within the sporangium formed in the anther locule. In *Arabidopsis*, the hypodermal cell in the sporangium enlarges to form the archesporial cell that then divides to generate the primary sporogenous cell toward the inside and the primary parietal cell in the outside. The sporogenous cell undergoes mitosis to give rise to PMCs, while the primary parietal cell divides to form the anther wall comprising the epidermis, the endothecium, the middle layer, and the tapetum (**Figure 1**, Maheshwari, 1950). Male sporogenesis is completed after meiosis resulting in four viable haploid microspores.

### CHROMATIN DYNAMICS DURING SMC DIFFERENTIATION

Here, we would like to review more particularly epigenetic events occurring and contributing locally to the somatic-to-reproductive transition taking place during sporogenesis. Specific chromatin dynamics related to meiotic execution will be described elsewhere in this issue (Plant Meiosis – Global Approaches).

**FIGURE 2:**
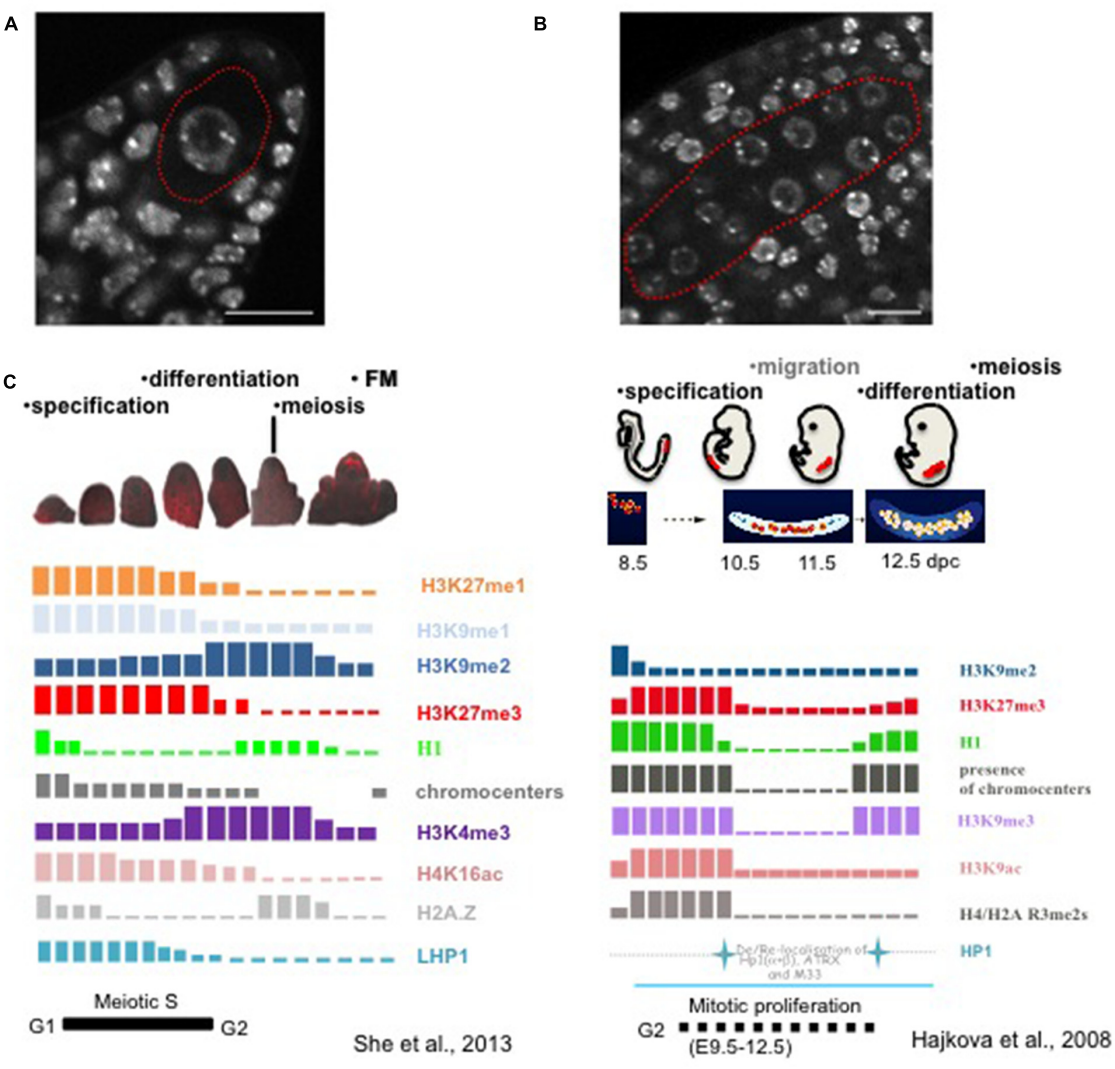
**Chromatin dynamics in plant MMCs shows similarities to that in animal PGCs. (A)** The MMC (red contour) originates from a subepidermal somatic cell in the ovule primordium, it is distinct from the surrounding nucellar cells by its enlarged nuclear size, as shown by whole-mount DNA staining using propidium iodide of the early ovule primordium as described (She et al., 2013). Scale bar: 10 μm. **(B)** Specification of PMCs (red contour) in the anther, which are marked by the enlarged nuclear and nucleolar size compared to the surrounding somatic cells. The anther was stained by propidium iodide in whole-mount as described for ovule primordia (She et al., 2013). Scale bar: 10 μm. **(C)** Likewise in animal PGCs, plant MMCs undergo drastic changes in chromatin modification patterns. The schemes summarize studies from Hajkova et al. (2008) and She et al. (2013). However and in contrast, events are asynchronous in plant MMCs and are characterized by both gain and depletion of marks, while animal PGCs at stage 10.5 show a marked depletion of all marks analyzed (Reprinted by permission from Macmillan Publishers Ltd: Nature, Hajkova et al., 2008),© 2008 and Prof. Azim Surani (The Gurdon Institute, University of Cambridge). The schematic images for PGCs development were modified after Ohno et al. (2013).

The first visible signs of SMC differentiation are cellular and nuclear enlargement in the sporogenous tissue. Visible changes in nuclear morphology during MMC differentiation were reported on early drawings or micrographs with clear nuclear and nucleolar enlargement compared to the surrounding nucellar cells (Cooper, 1937; Schulz and Jensen, 1981; Armstrong and Jones, 2003; Sniezko, 2006). In light of our current understanding, these observations suggest large-scale chromatin reorganization. Nuclear swelling and chromatin decondensation in differentiating MMC was recently confirmed and quantified (**Figure 2A**, She et al., 2013). Interestingly, it correlates with the depletion of canonical linker histones and the concomitant, yet progressive reduction in heterochromatin content (She et al., 2013). This H1 depletion is the earliest event of MMC differentiation at a stage where cellular differentiation is barely visible strongly suggests a causal link between chromatin dynamics and the somatic-to-reproductive fate transition in this cell. Following this event, the MMC chromatin undergoes further nucleosome remodeling and biphasic changes in histone modifications (Figure 2C). Nucleosome remodeling is illustrated by a presumably dynamic turnover of the centromeric-specific H3 variant (CENH3). This was incidentally detected in the MMC by the depletion of a C-terminally tagged CENH3 variant that failed to be reloaded, in contrast to its N-terminally tagged counterpart (She et al., 2013), in agreement with the model established in male SMCs (Ravi et al., 2011; Schubert et al., 2014). Moreover, the incorporation of a specific H3.3 variant (HTR8) in the MMC suggests global changes in nucleosome composition. Further chromatin dynamics events affecting histone modifications occur along a long meiotic S-phase and seem to establish a transcriptionally permissive state (She et al., 2013). This is suggested by a quantitative increase in the permissive-associated mark H3K4me3, and the reduction of repressive-related marks including H3K27me1, H3K27me3, and H3K9me1 in MMCs, compared to that in surrounding nucellar cells (She et al., 2013). However, decreasing levels of Ser2-phosphorylated RNA PolII and H4Kac16 indicated a moderate transcriptional competence.

The events described in the MMC are reminiscent of those observed in mouse primordial germ cells (PGCs) that can be seen as functional equivalent of plant SMCs: mouse PGCs undergo large-scale chromatin reprogramming characterized by chromatin decondensation, DNA demethylation, depletion of linker histone, histone replacement, and extensive erasure of the histone marks such as H3K9me2, H3K9ac, H3K9me3 and H3K27me3 (**Figure 2C**, Hajkova et al., 2002, 2008).

Whether pre-meiotic reprogramming of the DNA methylation landscape occurs, in the MMC, remains a fundamental question to address. At least, genetic evidence showed that DNA methylation landscape influences meiotic recombination in *Arabidopsis* (Mirouze et al., 2012). Post-meiotic reprogramming has been suggested largely based on the expression dynamics of DNA methyltransferases in the female gametophyte (see Chromatin Dynamics During Female Gametogenesis). However, the specific impact on the actual gametic epigenome remains unknown. Possibly, given their mechanistic link with DNA methylation, H1 and H2A.Z depletion in the MMC may enable profound remodeling of the methylome already in the MMC (Wierzbicki and Jerzmanowski, 2005; Kumar and Wigge, 2010; Zemach et al., 2013). Resolving the genomic loci targeted by those epigenetic reprogramming events, at the DNA or histone modification level, is the next challenge to address. However, the techniques that would enable MMC-specific chromatin profiling are not yet established.

The mechanisms controlling chromatin reprogramming in the MMC are likely to be diverse, including both active and passive processes. For instance, proteasome-mediated degradation controls histone variants eviction such as H1 (She et al., 2013) and possibly H2A.Z too. Yet, upstream modifications such as phosphorylation, ubiquitinylation, or citrullination may contribute to destabilize these variants (Contreras et al., 2003; Christophorou et al., 2014). Furthermore, some changes in histone modifications may be coupled with replication occurring during meiotic S phase: the reduction in H3K27me3 levels (relative to the increasing DNA content) may be caused by incorporation of new, non-modified nucleosomes during DNA replication. This, however, does not hold true for marks such as H3K4me3 and H3K9me2 that do show a relative increase during MMC differentiation and are likely involving the activity of chromatin-modifying enzymes. Yet, the process may still be mechanistically coupled: it is noteworthy that H3K9me2 increases at chromocenters at stages where DNA replication is mostly detected in these domains while H3K4me3 increases in euchromatin at later stages where DNA replication is mostly detected in this nuclear compartment (She et al., 2013). Finally, we may speculate that part of the chromatin dynamics may be mediated *in trans* as suggested by the large representation of small-RNA silencing effectors in the MMC transcriptome (Schmidt et al., 2011).

In contrast, chromatin dynamics events underlying PMC differentiation in the anther are barely known. Yet, similar to MMCs, PMC nuclei enlarge in the male sporangium compared to the surrounding tapetum in different species (Maheshwari, 1950, Figure 2B). The finding that transposable elements become expressed in PMCs may further suggest decondensation at heterochromatin loci (Yang et al., 2011) like in MMCs. In addition, remodeling of the nucleosome composition is very likely to occur in PMC likewise in MMCs, as suggested by the dynamic turnover of the centromeric-specific H3 variant (CENH3) detected in both rye and *Arabidopsis* (Ravi et al., 2011; Schubert et al., 2014). H1 linker histones are dynamically phosphorylated – hence potentially destabilized – during the meiotic S-phase of wheat meiocytes (Greer et al., 2012), consistent with the observation of reduced levels in *Arabidopsis* PMCs (Célia Baroux, unpublished). It would be interesting to determine whether the PMC chromatin undergoes a selective replacement of histone H1 with a male-specific variant, possibly resembling that of mouse testis (Sasaki et al., 1990). Collectively, these observations suggest that large-scale, chromatin dynamics may operate PMC fate establishment similar to that in MMCs, but detailed investigations remain necessary to confirm this proposal.

### FUNCTIONS FOR CHROMATIN DYNAMICS IN THE SMCs

#### Preparation for meiosis

The differentiation of SMCs is followed by meiotic prophase I, with homologous chromosome pairing, synapsis, and recombination. In mice, H3K9me2 deposition is critical for synapsis and in yeast, H3K4me3 marks meiotic recombination initiation sites and regulates double-stranded DNA breaks (Tachibana et al., 2007; Borde et al., 2009; Kniewel and Keeney, 2009). H3K9me2 and H3K4me3 enrichment in the chromatin of plant MMCs during the meiotic S-phase but also during prophase I (She et al., 2013) may suggest a similar role for these marks in synapsis and recombination initiation. Furthermore, the role of DNA methylation in determining the recombination landscape in *Arabidopsis* meiocytes (Melamed-Bessudo and Levy, 2012; Mirouze et al., 2012) may be contributed by H1 and H2A.Z dynamics in the MMC, two histone variants shown to influence DNA methylation patterns in *Arabidopsis* (Wierzbicki and Jerzmanowski, 2005; Zemach et al., 2013). But whether these epigenetic marks directly instruct the meiotic machinery is not known. Alternatively, an intuitive interpretation of chromatin dynamics in the MMC is to enable the expression of meiotic genes and the repression of the mitotic pathway. For instance, it was recently proposed that the female meiotic gene *DMC1* (*DISRUPTED MEIOTIC cDNA1*) is repressed in somatic cells by ACTIN RELATED PROTEIN6 (ARP6), thought to belong to chromatin modulating complexes, possibly via H2A.Z deposition (Qin et al., 2014). This model and the reported expression of *DMC1* in MMC of ovule primordia at stage 2-II is consistent with the eviction of H2A.Z from the MMC chromatin that thus likely enables meiotic gene derepression (She et al., 2013). Similarly in yeast, H1 depletion is a prerequisite to activate meiotic effectors and in mouse oocytes, H3K27 demethylation at key developmental genes in mouse is also essential to meiotic progression (Agger et al., 2007; Bryant et al., 2012). Thus, global remodeling of the meiocyte chromatin likely favors meiotic gene expression. However, it may not be the sole function, since ameiotic *ago9* MMCs resume similar chromatin dynamics than meiotic MMCs (She et al., 2013).

#### Repression of the somatic program

The SMC fate is not inherited, but it is established locally within a niche of somatic cells in floral sex organs. Intuitively, SMC specification may thus require to exit the somatic program. It was formerly proposed that a globally, epigenetic repressive landscape is established in the nucellus that may favor this transition (reviewed in Baroux et al., 2011; Feng et al., 2013). Several lines of evidence suggest that small-RNA-mediated silencing mechanisms may contribute to this process. ARGONAUTE proteins are central players in microRNAs (miRNAs) and small-interfering RNAs (siRNAs) directed post-transcriptional gene silencing (PTGS) and RNA directed DNA methylation (Vaucheret, 2008). In rice, *MEL1* encodes an AGO protein specifically expressed in SMCs before meiosis. Most SMCs cannot complete sporogenesis and arrest at early meiosis in the loss-of-function mutant, suggesting that MEL1 is important for switching from a mitotic to a meiotic program, a prerequisite for the somatic-to-reproductive cell fate transition. Possibly as well, *MEL1* may contribute to repress other somatic features as *mel1* mutant PMCs harbor somatic type of mitochondria (Nonomura et al., 2007). In maize, AGO104 specifically accumulates in the nucellar cells of ovule primordium during sporogenesis. MMCs lacking *ago104* activity fail to undergo meiosis, resulting in unreduced (diploid) embryo sacs. Transcriptional profiling of the *ago104* mutant suggests that it represses somatic gene expression in a non-cell autonomous way (Singh et al., 2011). Collectively, the above studies allow to propose a small-RNA-mediated repression of the somatic cell fate during SMC specification. Interestingly, this situation is reminiscent of the animal germline which differentiation requires the inhibition of the somatic transcriptional program, partially relying on piwiRNA-mediated silencing (Nakamura et al., 2010). A non-coding RNA transcribed by the gene *polar granule component* (*pgc*) represses somatic gene expression in *Drosophila* germ cells (pole cells; Martinho et al., 2004).

#### TE silencing during sporogenesis?

Transmitting the genetic information to the next generation without accumulated mutations is a considerable challenge for sexually reproducing organisms. Transposable elements (TE) are potentially mobile sequences within the genome that pose a threat to genome integrity. Epigenetic reprogramming during germline formation in animals, during sporogenesis in plants, is a potential risky window for TE to escape silencing. Both plants and animals have evolved different strategies to restrict TE activity, particularly in the germline (reviewed in Bao and Yan, 2012, see Companion Cell-Dependent TE Silencing in the Gametes to Preserve Genome Integrity). Chromatin decondensation, loss of heterochromatin, and genome-wide remodeling of the epigenetic landscape during MMC, and likely PMC, specification in plants create a favorable environment for TE escape, thus control mechanisms are likely in place for restricting TE activity in these cells. In somatic plant cells, TEs are kept silenced via an RNA-dependent DNA Methylation (RdDM) pathway, with 24 nt long siRNA targeting DNA and H3K9 methylation at TE loci (Xu et al., 2013). In the MMC, despite a very low heterochromatin content (10.51% compared to 32.3% of somatic cells), the remaining chromocenters are highly enriched in H3K9me2 (She et al., 2013), whereby the immunostaining signals largely overcome the chromocenter foci. This suggests the possibility that TE silencing is reinforced although heterochromatin domains are not maintained. Furthermore, TE silencing could be mediated in trans by siRNAs produced by the surrounding, somatic cells of the nucellus (Olmedo-Monfil et al., 2010). Plants deficient in RdDM-mediated silencing are unable to exert a control on TE proliferation when the parental plant was subjected to heat stress and transmit novel TE copies to their progeny. Genetic analyses suggested that this control normally takes place in the floral tissue and not during gametogenesis (Ito et al., 2011). This heat-activated TEs proliferate during chromatin reprogramming in the MMC of RdDM-deficient nucellus respectively, is the most plausible explanation. Consistent with this, the transcriptionally activated retrotransposon, EVADE, was shown to be actively, maternally suppressed via an siRNA-mediated heterochromatin pathway before meiosis (Reinders et al., 2013) suggesting further a siRNA-based mechanism to doom TE activity during chromatin reprogramming in the MMC.

#### Epigenetic reprogramming toward pluripotency establishment

Sporogenesis achieves the formation of a haploid, pluripotent spore, which will generate several distinct cell types upon gametophyte development. It has been proposed that chromatin reprogramming in the MMC contributes to establish competence to the gametophytic, pluripotent development of the spore. This proposal is based on the analysis of mutants forming ectopic, ameiotic gametophytes in the ovule (*ago9*, Olmedo-Monfil et al., 2010) and the *sdg2* mutant that lost female gametophytic competence (Berr et al., 2010); in those mutants with altered gametophytic competence, chromatin dynamics was either ectopically expressed (H1 eviction, H3.3 incorporation, H3K27me1 and H3K27me3 reduction) or with altered H3K4me3 levels, respectively.

Although a systematic functional dissection and a challenging, single-cell epigenome profiling remain to be done to confirm this hypothesis, large-scale chromatin dynamics in the MMC likely enables reprogramming the epigenetic landscape to prime a gametophytic developmental program. This situation is also highly reminiscent of that in mice where epigenetic reprogramming in PGCs establishes a ground-state epigenome and alleviates barriers against pluripotency in the germline (Yamaji et al., 2008; Hajkova, 2011; Hackett et al., 2012). Specifically, it would be interesting to test whether H3K27 demethylation in the MMC underlies transcriptional derepression of gametophytic genes, similar to the derepression of pluripotency genes in mice and humans, mediated by the H3K27 demethylase Utx (Mansour et al., 2012). The only H3K27 demethylase characterized so far in *Arabidopsis*, REF6 (Lu et al., 2011) does not seem to be involved in this process (She et al., 2013); thus determining the possible role of H3K27me3 on gametophytic gene expression awaits the elucidation of the mechanisms by which the MMC chromatin is depleted of H3K27me3.

## CHROMATIN DYNAMICS DURING GAMETOGENESIS

In plants, gametogenesis is the last step of gametophyte development. The gametes are differentiated, together with accessory cells, within the multicellular male and female gametophytes. In both cases, the establishment of distinct cell fates from genetically identical haploid cells is underlined by distinct chromatin organization.

### CHROMATIN DYNAMICS DURING MALE GAMETOGENESIS

**FIGURE 3:**
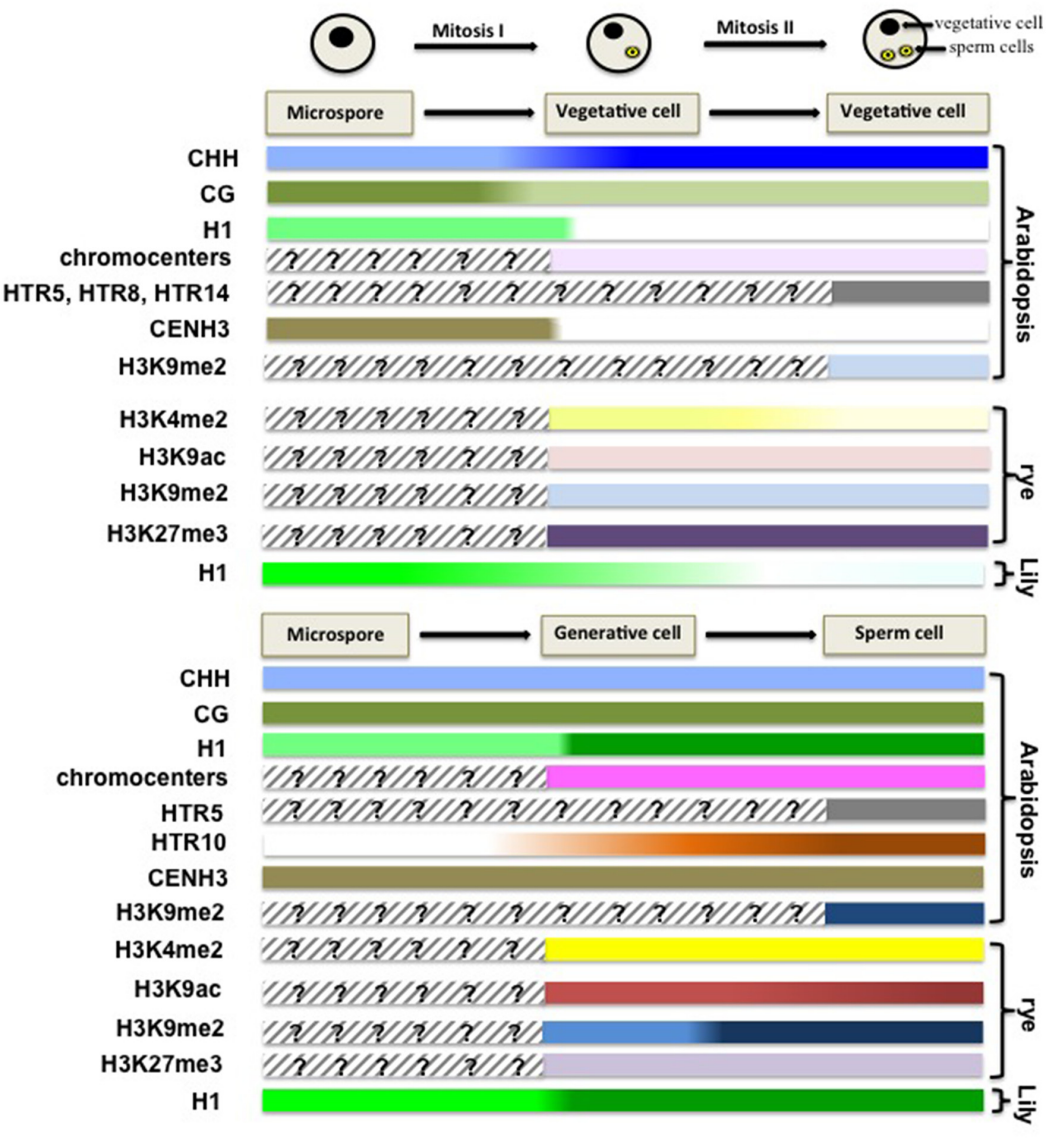
**Chromatin dynamics during male gametogenesis.** This scheme summarizes cytogenetic and molecular profiling data suggesting large-scale chromatin dynamics events during male gametophyte development. Although disparate in the level of investigation and plant species analyzed it provides a conceptual framework, yet to be completed, for apprehending the extent and potential significance of chromatin dynamics during this developmental stage. In *Arabidopsis*, the microspore harbors low levels of CHH methylation at retrotransposon loci, but retains CG methylation. After the first mitosis, the vegetative nucleus restores CHH methylation, but undergoes CG demethylation at a subset of TE loci (Calarco et al., 2012). The chromatin of the vegetative cell is highly decondensed, mostly deprived of linker H1 (Wenjing She and Célia Baroux, unpublished) and H3K9me2 (Schoft et al., 2009). Additionally, the somatic patterns of histone H3 variants are erased, and only a few H3 variants are retained including HTR5, HTR8, and HTR14 (Ingouff et al., 2010). Compared to that in somatic nuclei, the chromatin of vegetative cell in rye lost H3K4me2, H3K9ac and H3K9me2, but retains H3K27me3, which can be traced back to the bicellular stage (Houben et al., 2011). In contrast, the sperm chromatin inherits the pattern of DNA methylation from the microspore nucleus, with low levels of CHH methylation, and enrichment of methylated CG (Calarco et al., 2012). It accumulates linker histone H1.1 (Wenjing She and Célia Baroux, unpublished) and H3K9me2 (Schoft et al., 2009). Dynamic changes in the histone H3 repertoire are also observed, with erasure of the somatic variants, but enrichment in HTR5, HTR10 in the sperm nucleus (Ingouff et al., 2010). In rye, it was shown that the sperm chromatin is enriched in H3K4me2, H3K9ac and H3K9me2 modifications, but depleted of H3K27me3, a state that can be traced back to the generative cell at the bicellular stage (Houben et al., 2011).

Microgametogenesis begins with an asymmetric and atypical mitosis in the microspore, resulting in the formation of a large vegetative cell engulfing a smaller generative cell in *Arabidopsis*. The vegetative cell arrests at G1-phase, while the generative cell undergoes another mitosis to produce two sperm cells (Berger and Twell, 2011). The vegetative cell serves the function of delivering the male gametes toward the ovule during fertilization. The structurally and functionally different cell types are also marked by their dimorphic chromatin states (**Figure 3**).

The chromatin of the vegetative cell is largely decondensed compared to that of the somatic cells, with, notably, low levels of H3K9me2 in both eudicots and monocot species (Schoft et al., 2009; Houben et al., 2011). In *Arabidopsis*, the observed disperse of 180-bp centromeric repeats (180CEN) is possibly caused by the absence of the chromatin remodeler DDM1 (DECREASE IN DNA METHYLATION 1) from the SWI/SNF-family of in this cell (Probst et al., 2003; Schoft et al., 2009). Likely as a consequence of this chromatin state, massive transcription of transposable elements (TE) is observed, generating in turn TE-specific small-RNAs (Slotkin et al., 2009). While chromatin decondensation and depletion of repressive chromatin marks such as H3K9me2 likely favors active transcription, low levels of H3K4me2 and H3K9ac, two permissive marks, at least in rye, suggests that transcriptional competence is established independently of these usual modifications (**Figure 3**, Houben et al., 2011).

In contrast to the vegetative cell, the chromatin of the sperm cell is highly condensed. There, transcriptional activity is almost undetectable, based on immunolocalization of Ser2-P-PolII (Houben et al., 2011), although a large amount of transcripts are detected (Borges et al., 2008). This landscape may be partly contributed by high H3K9me2 levels, particularly at heterochromatin loci. However, and paradoxically, the sperm chromatin is enriched in H3K4me2 and H3K9ac, two transcriptionally permissive marks, while globally depleted in the repressive mark H3K27me3 (**Figure 3**, Houben et al., 2011). Collectively, these observations could suggest that the sperm chromatin acquires a poised state as in the animal germline.

Male gametogenesis is also accompanied by changes in the histone H3 variant repertoire, with distinct patterns established between the sperm and the vegetative cells, which can be observed early at the bicellular stage (**Figure 3**). While both cells are devoid of the somatic H3.1 variants, they contain each a specific repertoire of H3.3 variants: the chromatin of the vegetative cell includes a few canonical H3.3 variants (HTR5 and HTR8) and the variant HTR14, while the sperm chromatin contains HTR5 and a sperm-specific variant (HTR10; Ingouff et al., 2010). Dynamics of core histone variants is also described in Lily pollen, with the specific incorporation in the generative cell of gH2A, gH2B, gH3 – which shares common structural properties with Arabidospsis CENH3 – and the selective depletion of somatic H1 in the vegetative cells (Tanaka et al., 1998; Xu et al., 1999; Ueda et al., 2000).

Chromatin dynamics during male gametophyte development is also reflected by the distinct DNA methylation patterns established between the vegetative cell and the gametes, which can be traced back to the microspore stage before mitosis I (**Figure 3**). Comparatively to somatic cells, the microspore chromatin is devoid of CHH methylation mostly from retrotransposon loci. Gametogenesis entails antagonist changes in the sperm and vegetative cells: while the sperm cells inherit the CHH DNA methylation patterns from the microspore, with more pronounced depletion, the vegetative cells restore CHH methylation at TE loci. In contrast, CG methylation is globally retained, in the sperm cells, but depleted from a subset of TE loci and intergenic regions in the vegetative cell. While compared to that in the sperm cells, CHG methylation is generally higher in the vegetative cell, albeit depleted from the same demethylated CG TE loci (Calarco et al., 2012; Ibarra et al., 2012). This profound, dimorphic remodeling of DNA methylomes during microgametogenesis is likely a consequence of differential activity of key factors in the gametes and vegetative cell: the de novo DNA methyltransferase DRM2 and the 24nt siRNA-based machinery, that normally act together in establishing and maintaining CHH methylation, respectively, and the DNA glycosylases DEMETER (DME) and REPRESSOR OF SILENCING 1 (ROS1) enabling CG demethylation via a base-pair excision-repair process (Morales-Ruiz et al., 2006; Law and Jacobsen, 2010; Calarco et al., 2012).

Whether DNA methylome reprogramming is a cause or consequence of large-scale chromatin dynamics is unclear. Possibly, however, depletion of H1 linker histones and of the chromatin remodeler DDM1 in the microspores (Tanaka et al., 1998; Wenjing She and Célia Baroux, unpublished) may underscore a mechanistic link with DNA methylation changes (Wierzbicki and Jerzmanowski, 2005; Zemach et al., 2013).

### CHROMATIN DYNAMICS DURING FEMALE GAMETOGENESIS

**FIGURE 4:**
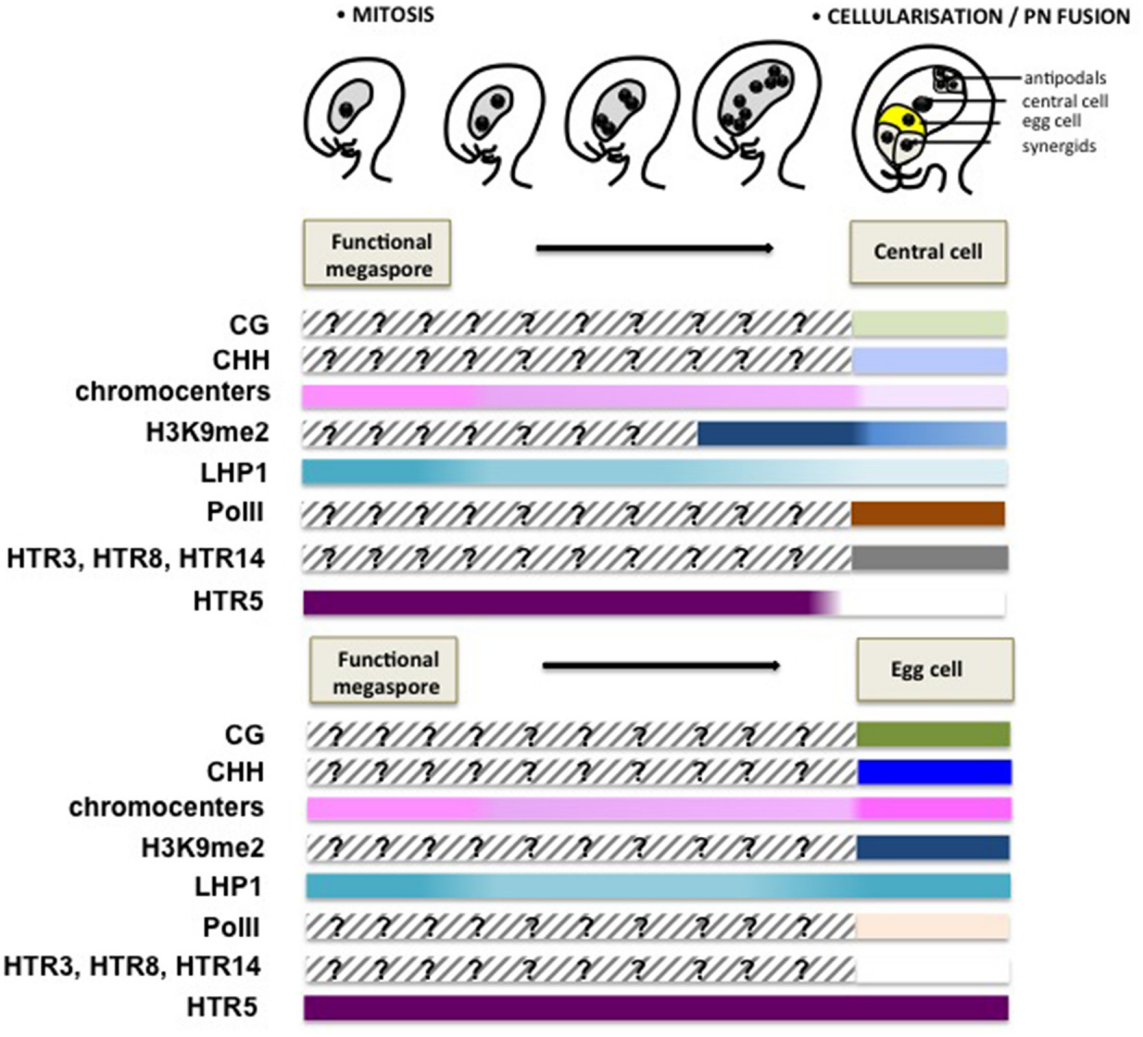
**Chromatin dynamics during female gametogenesis.** This scheme summarizes mostly cytogenetic and GFP reporter protein analyses suggesting large-scale chromatin dynamics events during female gametophyte development. Although genome-wide, molecular profiling of the chromatin state is currently missing, these data provide, like for **Figure 3**, a conceptual framework for apprehending the extent and potential significance of chromatin dynamics during this developmental stage. Following cellularization, a dimorphic chromatin landscapes are established between the egg cell and the central cell. The central cell chromatin harbors a decondensed chromatin with a low heterochromatin content, correlating with low levels of H3K9me2 and the H3K27me3 reader protein LHP1, but is enriched in active PolII (Ser2 phosphorylated PolII) allowing for active transcription (Pillot et al., 2010). The notable absence of DNA methyltransferases and the presence of the DNA glycosylase DEMETER catalyzing DNA methylation suggest a hypomethylated genome. In contrast, the egg cell harbors heterochromatin foci, though not as prominently as in somatic nuclei and high levels of H3K9me2 and LHP1, but undetectable levels of PolII, suggesting a repressed transcriptional state. Somatic histone variants are depleted from both gametes, with only HTR3, HTR8 and HTR14 retained in the central cell and HTR5 in the egg cell. The model for dynamic changes of CG and CHH methylation is speculative, and is inferred from the analysis of DNA methylation in the endosperm and embryo (Hsieh et al., 2009; Ibarra et al., 2012), as well as the differential expression of DNA methyltransferases between the central cell and egg cell (Jullien et al., 2012). The epigenetic dimorphism concerning heterochromatin content, H3K9me2 and LHP1 seems established just after cellularization.

The female gametophyte has a syncytial mode of development until the eight-nuclear stage. The bipolar organization of the gametophyte is short lived and migration of two polar nuclei toward the center of the syncytium quickly sets the future pattern of the mature embryo sac, which is definitively set at cellularization (Sprunck and Gross-Hardt, 2011). A microscopic observation of the nuclear size and chromatin appearance at the consecutive stages of development suggests a rather decondensed state of the chromatin but also rapid changes entailed by cellularization (Célia Baroux, unpublished). Particularly, while the antipodals and synergids seem to regain a chromatin organization similar to that of sporophytic cells, the egg and the central cells reveal globally less condensed chromatin state, with fewer heterochromatin foci compared to that of the somatic cells (Jullien and Berger, 2010; Baroux et al., 2011). Yet, the gametes appear clearly dimorphic with a more pronounced decondensation in the central cell and this dimorphism, similar to that between the vegetative cell and the sperm cells, respectively, in the male gametophyte, is further illustrated by the distinct epigenetic and transcriptional landscapes detected using cytogenetic investigations (Pillot et al., 2010). The chromatin in the central cell shows a dramatic reduction of H3K9me2 and LHP1 induced at/after cellularization of the gametophyte, while being transcriptionally active. In contrast, the egg cell chromatin harbors high levels of LHP1 and H3K9me2 at conspicuous foci, coincidentally with low-to-undetectable levels of active RNA PolII, reflecting a relatively transcriptional quiescent state (Pillot et al., 2010; **Figure 4**). Concomitantly, unequal expression of DNA methyltransferases in the central cell and egg cell – with notably undetectable level of these enzymes in the central cell contrasting with the presence of *de novo* DNA methyltransferases DRM1/2 in the egg – may contribute to reinforce the epigenetic dimorphism (Jullien et al., 2012).

The dimorphic epigenetic state between the egg cell and the central cell is also reflected by the establishment of distinct core histone variant patterns (**Figure 4**). Similar to that in the male gametes, both of the female gametes are devoid of most of the canonical, somatic H3 variants. The mature egg cell only harbors the H3.3 variant HTR5, while the central cell retains one H3.1 (HTR3) and two H3.3 variants (HTR8 and HTR14; Ingouff et al., 2010; **Figure 4**). It was considered that the absence of H3.1 in the egg cell may be caused by the arrested cell cycle before S-phase, as H3.1 incorporation is linked with DNA synthesis (Ingouff et al., 2010; Stroud et al., 2012). The specific eviction of core histone H2B in the egg cell, rather than in the central cell, further underlines dimorphic chromatin composition between the gametes (Pillot et al., 2010).

In addition, compared to that in the egg cell where low levels of maintenance DNA methyltransferases including MET1 and CMT3, and high levels of *de novo* DNA methyltransferases (DRM1/2) are detected, the central cell keeps barely detectable levels of MET1 and CMT3 and low levels of DRM1/2 (Jullien et al., 2012), where *MET1* was proposed to be repressed in the central cell via a Retinoblastoma pathway (Jullien et al., 2008). Furthermore, the DNA demethylase DME is specifically expressed in the central cell, but not in the egg cell prior to fertilization (Choi et al., 2002). Differential expression of those enzymes suggests that the central cell has a globally hypomethylated genome compared to the egg cell (**Figure 4**). While this model is often taken for granted largely due to inferences made from DNA methylome profiling data in the fertilization products at a relatively late stage of seed development (Gehring et al., 2009; Hsieh et al., 2009; Zemach et al., 2010), probing the genome for effective DNA methylation, in sequence context, using cytogenetic and molecular profiling approaches remain necessary to confirm the quantitative and qualitative distinction between the female gametes. In addition, the possibility remains that some loci may be preferentially demethylated after fertilization rather than in the central cell (Jahnke and Scholten, 2009). While instances of hypomethylated genes in the central cell could be described for a few loci in isolated maize gametes (Gutierrez-Marcos et al., 2006; Jahnke and Scholten, 2009), genome-wide profiling of the DNA methylomes, and histone modifications, specifically in the egg and central cells remains currently an immense challenge, due to the extreme difficulty in isolating those cells at a large scale.

### FUNCTIONS OF CHROMATIN DYNAMICS DURING GAMETOGENESIS

#### Derepression of gametic-specific genes

Both the female and male gamete transcriptomes are characterized by a set of specific expressed genes that are otherwise silent in somatic tissues (Wuest et al., 2010; Russell et al., 2012). A few examples report on a contribution of chromatin-mediated repression in this process: for instance, some male-gamete-specific genes were found to be actively repressed by H3K27me1 and H3K27me3 in the sporophyte (Hoffmann and Palmgren, 2013). Thus, chromatin dynamics occurring during gametogenesis and achieving cell-specific epigenetic landscapes (see above) may create a favorable environment for the derepression of those gamete-specific genes. To investigate this hypothesis, it would be of interest to monitor the precise timing of gamete-specific gene expression in relation to the chromatin dynamics events reported above.

#### Companion cell-dependent TE silencing in the gametes to preserve genome integrity

The problem of maintaining genome integrity in the germline has been exposed in section TE silencing during sporogenesis. In mice, the requirement of a TE control in the germline is restricted to ***PGC*** development and meiosis (Bao and Yan, 2012), since the meiotic product directly produces the mature gamete. In plants, however, the mitotic developmental phase of the gametophyte, following meiosis, imposes the necessity to prolong a control over TE activity until the mature gametes.

Unlike the sperm cells, the vegetative cell does not contribute to the next generation. Yet, this companion cell seems to influence the epigenetic setup of the sperm cells. The current model involves TE-derived 21nt siRNAs produced by the vegetative cell (following passive and active DNA demethylation) that act in trans on the sperm cells’ chromatin to reinforce TE silencing via RNA-directed DNA methylation (RdDM; Slotkin et al., 2009; Ibarra et al., 2012). The efficient silencing of a GFP reporter gene in the sperm cells by expressing the corresponding artificial microRNA in the vegetative cell under the LAT52 promoter supports the model of small RNA transfer from the companion cell to the male gametes (Slotkin et al., 2009; Feng et al., 2013). However, in another study using a promoter specifically activated in the vegetative cell and not at earlier stage of microspore development (unlike LAT52) this trans silencing experiment could not be reproduced suggesting that TEs siRNAs in the sperm cells may be inherited from the microspore (Grant-Downton et al., 2013). Thus, although further analysis is needed, the model prevails that the companion cell provides a process of genome integrity maintenance in sperm cells that are transcriptionally silent and thus unable to provide the effectors for TE silencing.

Likewise in sperm cells, a control over TE activity in the egg cell would be meaningful. It has been proposed that, similar to the vegetative cell toward the sperm cells, the central cell may play a role in reinforcing TE silencing in the egg cell. This model is inferred from the observation that when the endosperm is derived from a central cell lacking the activity of the DEMETER DNA glycosylase, hypermethylation of TEs is observed, suggesting that those loci are normally demethylated (by DME) and may, likewise the vegetative cell produces TE-derived siRNA. Similarly, trans-silencing of a reporter gene was successfully achieved in the egg by expressing the corresponding amiRNA in the central cell (Ibarra et al., 2012), comforting the idea that siRNA transpose from the central cell to the egg cell to maintain genome integrity in the female germline too (reviewed in Feng et al., 2013).

#### Setting epigenetic asymmetry for genomic imprinting

Genomic imprinting refers to epigenetic regulations leading to unequal expression of both parental alleles in a diploid cell, thereby conveying possible parent-of-origin-specific effects at the molecular, cellular, tissue, or organismal level. In plants, imprinting occurs in both the embryo and endosperm (reviewed in Dickinson and Scholten, 2013; Gehring, 2013). Genetic studies indicated that imprinting regulation involves differentially methylated regions (DMRs) but also PRC2-mediated histone modifications and likely other, yet unknown, epigenetic mechanisms (Gehring, 2013). The mechanisms of imprinting regulation are extensively reviewed elsewhere (e.g., Raissig et al., 2011; Köhler et al., 2012; Dickinson and Scholten, 2013; Gehring, 2013) and will not be treated in detail here. However, it is relevant to outline the basic principle that imprinting regulation relies on an asymmetric epigenetic setup between the parental alleles that has to be established prior to fertilization. So far, the current model suggests that the parental alleles are, by default, set in an epigenetically repressed state inherited from the somatic cells while a gender-specific erasure of a, e.g., silencing mark enables priming expression after fertilization. For instance, maternally expressed genes *(MEG)* active in the endosperm are demethylated in the central cell via both active and passive mechanisms (DME-mediated DNA demethylation and lack of DNA methylation maintenance by MET1, respectively, Jullien and Berger, 2010), while their paternal counterpart are hypermethylated in the sperm cells. This is the case for instance for *FLOWERING WAGENINGEN* (*FWA*), *FERTILIZATION INDEPENDENT SEEDS2* (*FIS2*), *MEDEA (MEA*; see reviews cited above), although the latter can be maternally activated in a DME and DNA-methylation-independent manner (Wohrmann et al., 2012). Interestingly, the absence of DME in conjunction with the presence of *de novo* DNA methyltransferases in the egg cell, together with genetic studies on embryo-*MEG* regulation, suggests that the establishment of imprints to be inherited to the embryo relies on distinct mechanisms (Dickinson and Scholten, 2013; Raissig et al., 2013a). The wide range of chromatin changes, including male- or female-gamete-specific resetting of the histone H3 repertoire, and possibly of other histone variants, may offer alternative means to asymmetrically mark the parental alleles of imprinted loci.

Interestingly, gametogenesis is the sole developmental window considered so far for establishing the epigenetic setup of imprinted loci. However, sporogenesis, more particularly SMC differentiation that undergoes massive reprogramming of its chromatin landscape (see Chromatin Dynamics During Sporogenesis) offers another window of opportunity to establish parental imprints. Erasure of DNA methylation at MEG loci for instance may be achieved in the MMC following the eviction of H1 and H2AZ (She et al., 2013) known for their interplay with DNA methylation (Wierzbicki and Jerzmanowski, 2005; Rea et al., 2012; Zemach et al., 2013).

#### Pre-patterning the post-fertilization fates

The distinct chromatin states established in the egg cell and the central cell after cellularization of the female gametophyte reflect distinct epigenetic and transcriptional status. An interesting explanation for the transcriptionally quiescent state of the egg cell may be a role for establishing totipotency in the zygote likewise in animals. In animals, the zygote is transcriptionally inactive for a duration that varies depending on the species; this transient status (preceding zygotic genome activation) is thought to be necessary to epigenetically reprogram the zygotic genome toward a totipotent ability, through priming developmental regulator genes for expression (Seydoux and Braun, 2006; Surani et al., 2007). The case of plants may mirror that of the animals whereby chromatin dynamics in the egg may pattern the transcriptionally quiescent chromatin of the future zygote. In stark contrast to the egg, the central cell is epigenetically relaxed toward a highly permissive state and *de facto* transcriptionally active (Pillot et al., 2010), a state largely inherited in the endosperm following fertilization. Thus again here, chromatin dynamics in the central cell is likely a pre-patterning event of its post-fertilization fate. What are the critical epigenetic remodeling events that contribute to the identity of the gametes themselves and their post-fertilization products is however still unknown. Clearly, transcriptional quiescence is not enough to define a totipotent state, since an artificially induced transcriptionally silent state in the central cell results in abortion of its post-fertilization program (the endosperm fails to develop; Pillot et al., 2010). Conversely, mutant zygotes deficient in RdDM-mediated gene silencing are transcriptionally active, yet developmentally competent to form a viable embryo (Autran et al., 2011). Being able to profile the epigenetic landscape, genome-wide and at single-gene resolution is critically required to decipher the targets and role of chromatin dynamics in the gametes.

## CHROMATIN DYNAMICS FOLLOWING DOUBLE FERTILIZATION

### DIMORPHIC CHROMATIN LANDSCAPES ESTABLISHED IN TWO FERTILIZATION PRODUCTS

Embryogenesis is a long developmental process progressing along consecutive phases of proliferation, morphogenesis, organogenesis, and maturation. Our knowledge is too scarce to draw a developmental atlas of chromatin dynamics events during those phases; large-scale processes have been mostly reported both immediately following fertilization, on which we will focus below, or at maturation stages (van Zanten et al., 2011).

Soon after fertilization, rapid exchanges of gametic H3.3 histone variants occur in the zygote and endosperm and a somatic pattern of H3.3 variant composition is reestablished in the zygote (Ingouff et al., 2007, 2010). This suggests a limited inheritance of H3-based epigenetic information from the gametes to the fertilization products. Yet, the modest resolution of microscopic investigations does not allow excluding inheritance for discrete loci or small chromosomal segments. Clearly, however, the transcriptional states of the fertilized products are largely inherited from their female gametic progenitor: the zygote seems transcriptionally quiescent with barely detectable PolII activity while the endosperm harbors a transcriptionally active chromatin state as shown by abundant levels of engaged RNA PolII (Pillot et al., 2010). Additionally, the dimorphic pattern of H3K9me2 (high in the zygote, low in the endosperm) is also similar to that in the female gametes suggesting inheritance of at least some levels of chromatin organization. The functional requirement of the DNA methyltransferase CMT3 further suggests a connective interplay between H3K9 and DNA methylation in establishing this dimorphism (Pillot et al., 2010).

Furthermore, developmental progression of the fertilization products entails additional chromatin dynamics as inferred by the molecular profiles of DNA methylation patterns in the embryo and endosperm in well-developed seeds (6–8 days after pollination). Particularly, the maternal genome of the endosperm undergoes DME-mediated global DNA demethylation, while, comparatively, DNA methylation levels are higher in embryo in all sequence context (Hsieh et al., 2009). This dimorphism is consistent with the antagonist abundance of DNA methyltransferases including MET1, DRM2, and CMT3 in the embryo and endosperm, respectively (Hsieh et al., 2009; Jullien et al., 2012). The detection of those DNA methyltransferases in the embryo proper at early stage suggests the hypothesis that reprogramming of the DNA methylation landscape already occurs soon after fertilization, likewise in mice (Seisenberger et al., 2013).

Overall, while several evidence suggest dynamic reprogramming of the chromatin states (histone and DNA modifications) in the fertilization products, the data remain uneven with distinct developmental stages investigated (e.g., profiles at early developmental stages are missing) and at different resolution levels (molecular profiles versus microscopic detection of immunostaining signals or fluorescently tagged chromatin modifiers). Clearly, temporally resolved profiles of histone and DNA methylation patterns in the developing embryo and endosperm starting from soon after fertilization is necessary to elucidate the epigenetic landscape and its dynamics. But for this, daunting technical difficulties remain to be solved to enable massive, and tissue specific nuclei isolation suited for epigenome profiling, particularly from the embryo and endosperm – that are embedded within the maternal seed – at very early stages.

### FUNCTIONS OF CHROMATIN DYNAMICS IN THE FERTILIZATION PRODUCTS

#### Reprogramming toward totipotency acquisition in the zygote?

Fertilization unites two differentiated and very specialized cells, the egg cell and the sperm cell. To enable embryo development-with the establishment of novel cell types and organ symmetries toward the basic body plan of the future plant – the newly formed zygote must be alleviated from the gametic programs and acquire totipotency. With analogy to animals, it is tempting to speculate that epigenetic reprogramming may occur in the plant zygote toward setting the future transcriptional program. The abrupt replacement of histone H3 variants in the zygote (Ingouff et al., 2010) may enable a rapid resetting of histone modifications toward this goal. However, we are currently lacking detailed molecular profiles in the gametes and the zygote to draw any meaningful comparison. Yet, strikingly, the plant zygote remains transcriptionally relatively quiescent (Pillot et al., 2010), a situation reminiscent to that of animal zygotes and which is necessary to totipotency acquisition (reviewed in Seydoux and Braun, 2006). Future efforts in elucidating and manipulating epigenome dynamics in the plant zygote are necessary, yet extremely challenging, to conclude about possible evolutionary convergent scenarios between the two kingdoms in the role of epigenetic reprogramming in totipotency acquisition.

#### Genomic imprinting

Imprinting in the embryo suggests the existence of mechanisms – yet to be discovered – enabling resistance at specific loci against the proposed genome-wide reprogramming of DNA methylation and histone modification landscapes. Imprinted loci seem to have, however, a shorter lifetime in the plant embryo than in animals. In contrast to animals where imprinting persists in adult tissues, no imprinted expression has been detected to date in the seedling which strongly suggests an erasure process of imprints at late stage of embryo development (Raissig et al., 2013a). The suggested, active remethylation of the embryo genome via the DNA methyltransferases DRM1/DRM2 (Jullien and Berger, 2010) may be important in this process (Jullien et al., 2012). Clearly, temporally resolved, DNA methylome profiles of the embryonic genome are awaited to comprehend the timing, the extent and the nature of loci affected by DNA methylation reprogramming following fertilization. While still challenging to perform, recent methodological progress in *Arabidopsis* embryo isolation and bisulfite sequencing from small input fractions offers the realistic possibility in a near future (Schmidt et al., 2012; Raissig et al., 2013b). In addition, the active maintenance of asymmetric histone modifications set by the PRC2 complex is necessary to perpetuate imprinting at several embryo imprinted loci (Raissig et al., 2013a), yet is in apparent contradiction with the global eviction of maternal H3 variants in the zygote (Ingouff et al., 2010). Thus clearly, chromatin dynamics cannot be described only globally but has to be resolved at the gene-specific level to understand its role in imprinting regulation in the embryo.

#### TE control for genome integrity across generation

The maternal genome of *Arabidopsis* endosperm undergoes extensive CG demethylation at TE loci, which at least partially requires *DME* activity (Hsieh et al., 2009). Similar to the situation described in the companion cells of the pollen, it has been proposed that TEs from the central cell, and possibly the endosperm as well, may produce specific siRNAs that reinforce TE silencing in the zygote, thereby dooming those genomic elements potentially harmful for the genome integrity of the ensuing generations (Mosher et al., 2009; Ibarra et al., 2012). Although the mobility of siRNA from the endosperm to the embryo remains to be confirmed, it was reported that demethylation of endosperm maternal genome is accompanied by CHH hypermethylation of TEs in the embryo (Hsieh et al., 2009). An endosperm-driven control of genome integrity surveillance in the embryo is likely a conserved mechanism across flowering plants, with evidence reported in both eudicots and monocots (Mosher et al., 2009; Zemach et al., 2010).

## CONCLUSIONS AND FUTURE PROSPECTIVES

**FIGURE 5:**
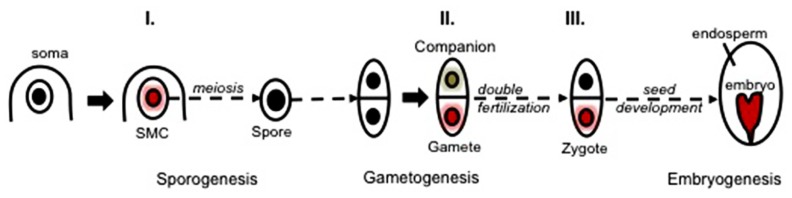
**Three main waves of chromatin dynamics during plant reproduction (Model).** Sexual plant reproduction can be seen as a three-step process involving sporogenesis, gametogenesis, and embryogenesis taking place in floral organs. Sporogenesis initiates with the specification of spore mother cells (SMCs) within the sporangium tissues. SMCs are primed toward meiosis while undergoing a somatic-to-reproductive cellular fate transition that generates a pluripotent spore. The spore develops a (male or female) multicellular gametophyte generating distinct cell types: the companion (or accessory) cells and the gametic cells (a schematically reduced form is shown, for more details see **Figure 1**). Fertilization enables the formation of a totipotent zygote, generating in turn the plant embryo. The acquisition of the SMC fate, the gametic fate and the totipotent zygotic fate is associated with three main waves of chromatin dynamics (I.–III., colored nuclei) comprising large-scale reorganization of the chromatin structure, composition and organization, hence reshaping the epigenetic landscape (as reviewed in the text). Whereas some of those events clearly contribute to cell fate establishment (e.g., I., see the text), the challenge of future investigations is to elucidate the functional role of chromatin dynamics in defining the cells’ potency versus operating cell fate establishment during sexual reproduction.

To date, a broad range of genetic and molecular regulators have been identified that contribute to cell specification processes during sexual reproduction in flowering plants. Yet, with the increasing body of evidence that these processes are accompanied by large-scale chromatin dynamics events, an exciting area is opening; further efforts are needed to comprehend a yet, underestimated level of control mediated by chromatin dynamics likely potentiating the (re)programming of genome expression during those processes. Exciting findings in the past decades uncovered dynamic events of chromatin modifications, DNA methylation, nucleosome remodeling, and small RNA regulation that take place throughout sexual reproduction in flowering plants, particularly during cell fate specification (**Figure 5**). The possible functions of these events range from epigenetic reprogramming of the genome toward pluri- or totipotency, maintenance of genome integrity, regulation of imprinting but may also functions in immediate cellular tasks at meiosis, mitosis cellularization and patterning in the gametophyte and embryo. In the absence of cell-specific epigenome profiles, however, the impact of chromatin dynamics on epigenetic reprogramming remains largely speculative. Establishing a dogma still requires efforts to overcome the daunting obstacles that obstruct cell-specific epigenome profiling in the reproductive lineage, particularly in the model plant *Arabidopsis thaliana*. For these experiments, the choice of other model plants (e.g., model crops) where the gametes are more amenable to mechanical isolation may be judicious. The development of cell-specific nuclei isolation approaches (Deal and Henikoff, 2010) may prove a real asset in these efforts, though it still requires improvement for optimization (Wuest et al., 2013). Alternatively, probing the genome at the microscopic scale for its chromatin composition and organization, at high-resolution, at the single-cell level and in a quantitative manner, has proven a valid and fruitful approach (She et al., 2014). It enabled describing unsuspected chromatin dynamics events during SMC and female gamete specification and, in combination with genetic analyses, revealed a functional link with the acquisition of developmental competences (Pillot et al., 2010; She et al., 2013). The completion of such analyses on the male reproductive lineage, in several (model or non-model plants) will be instrumental in determining whether cell specification during reproduction relies on robust, reiterative chromatin dynamics events across developmental phases and genders, and whether an evolutionary conserved scenario exists across eudicots and monocots.

## Conflict of Interest Statement

The authors declare that the research was conducted in the absence of any commercial or financial relationships that could be construed as a potential conflict of interest.

## References

[B1] Agger K. Cloos P. A. Christensen J. Pasini D. Rose S. Rappsilber J. (2007). UTX and JMJD3 are histone H3K27 demethylases involved in HOX gene regulation and development. *Nature* 449 731–734 10.1038/nature06145 17713478

[B2] Andrey P. Kieu K. Kress C. Lehmann G. Tirichine L. Liu Z. (2010). Statistical analysis of 3D images detects regular spatial distributions of centromeres and chromocenters in animal and plant nuclei. *PLoS Comput. Biol.* 6:e1000853 10.1371/journal.pcbi.1000853 PMC2900307 20628576

[B3] Armstrong S. J. Jones G. H. (2003). Meiotic cytology and chromosome behaviour in wild-type *Arabidopsis thaliana*. *J. Exp. Bot.* 54 1–10 10.1093/jxb/erg034 12456750

[B4] Autran D. Baroux C. Raissig M. T. Lenormand T. Wittig M. Grob S. (2011). Maternal epigenetic pathways control parental contributions to *Arabidopsis* early embryogenesis. *Cell* 145 707–719 10.1016/j.cell.2011.04.014 21620136

[B5] Bao J. Yan W. (2012). Male germline control of transposable elements. *Biol. Reprod.* 86:162 161–114 10.1095/biolreprod.111.095463 22357546 PMC3364930

[B6] Baroux C. Raissig M. T. Grossniklaus U. (2011). Epigenetic regulation and reprogramming during gamete formation in plants. *Curr. Opin. Genet. Dev* 21 124–133 10.1016/j.gde.2011.01.017 21324672

[B7] Berger F. Twell D. (2011). Germline specification and function in plants. *Annu. Rev. Plant Biol.* 62 461–484 10.1146/annurev-arplant-042110-103824 21332359

[B8] Berr A. Mccallum E. J. Menard R. Meyer D. Fuchs J. Dong A. (2010). Arabidopsis SET DOMAIN GROUP2 is required for H3K4 trimethylation and is crucial for both sporophyte and gametophyte development. *Plant Cell* 22 3232–3248 10.1105/tpc.110.079962 21037105 PMC2990135

[B9] Borde V. Robine N. Lin W. Bonfils S. Geli V. Nicolas A. (2009). Histone H3 lysine 4 trimethylation marks meiotic recombination initiation sites. *EMBO J.* 28 99–111 10.1038/emboj.2008.257 19078966 PMC2634730

[B10] Borges F. Gomes G. Gardner R. Moreno N. Mccormick S. Feijo J. A. (2008). Comparative transcriptomics of *Arabidopsis* sperm cells. *Plant Physiol.* 148 1168–1181 10.1104/pp.108.125229 18667720 PMC2556834

[B11] Bryant J. M. Govin J. Zhang L. Donahue G. Pugh B. F. Berger S. L. (2012). The linker histone plays a dual role during gametogenesis in *Saccharomyces* cerevisiae. *Mol. Cell. Biol.* 32 2771–2783 10.1128/MCB.00282-12 22586276 PMC3416202

[B12] Calarco J. P. Borges F. Donoghue M. T. Van Ex F. Jullien P. E. Lopes T. (2012). Reprogramming of DNA methylation in pollen guides epigenetic inheritance via small RNA. *Cell* 151 194–205 10.1016/j.cell.2012.09.001 23000270 PMC3697483

[B13] Chan S. W. Henderson I. R. Jacobsen S. E. (2005). Gardening the genome: DNA methylation in *Arabidopsis thaliana*. *Nat. Rev. Genet.* 6 351–360 10.1038/nrg1601 15861207

[B14] Choi Y. Gehring M. Johnson L. Hannon M. Harada J. J. Goldberg R. B. (2002). DEMETER, a DNA glycosylase domain protein, is required for endosperm gene imprinting and seed viability in *Arabidopsis*. *Cell* 110 33–42 10.1016/S0092-8674(02)00807-3 12150995

[B15] Christophorou M. A. Castelo-Branco G. Halley-Stott R. P. Oliveira C. S. Loos R. Radzisheuskaya A. (2014). Citrullination regulates pluripotency and histone H1 binding to chromatin. *Nature* 507 104–108 10.1038/nature12942 24463520 PMC4843970

[B16] Contreras A. Hale T. K. Stenoien D. L. Rosen J. M. Mancini M. A. Herrera R. E. (2003). The dynamic mobility of histone H1 Is regulated by cyclin/CDK phosphorylation. *Mol. Cell. Biol.* 23 8626–8636 10.1128/mcb.23.23.8626-8636.2003 14612406 PMC262667

[B17] Cooper D. C. (1937). Macrosporogenesis and embryo-sac development in euchlaena mexicana and zea mays. *J. Agric. Res.* 55 539–551

[B18] Costa L. M. Marshall E. Tesfaye M. Silverstein K. A. Mori M. Umetsu Y. (2014). Central cell-derived peptides regulate early embryo patterning in flowering plants. *Science* 344 168–172 10.1126/science.1243005 24723605

[B19] Deal R. B. Henikoff S. (2010). A simple method for gene expression and chromatin profiling of individual cell types within a tissue. *Dev. Cell* 18 1030–1040 10.1016/j.devcel.2010.05.013 20627084 PMC2905389

[B20] Dickinson H. Scholten S. (2013). And baby makes three: genomic imprinting in plant embryos. *PLoS Genet.* 9:e1003981 10.1371/journal.pgen.1003981 PMC3854966 24339794

[B21] Drews G. N. Koltunow A. M. (2011). The female gametophyte. *Arabidopsis Book* 9:e0155 10.1199/tab.0155 PMC3268550 22303279

[B22] Feng X. Zilberman D. Dickinson H. (2013). A conversation across generations: soma-germ cell crosstalk in plants. *Dev. Cell* 24 215–225 10.1016/j.devcel.2013.01.014 23410937

[B23] Fransz P. de Jong J. H. (2002). Chromatin dynamics in plants. *Curr. Opin. Plant Biol.* 5 560–567 10.1016/S1369-5266(02)00298-4 12393020

[B24] Fransz P. Ten Hoopen R. Tessadori F. (2006). Composition and formation of heterochromatin in *Arabidopsis thaliana*. *Chromosome Res.* 14 71–82 10.1007/s10577-005-1022-5 16506097

[B25] Gehring M. (2013). Genomic imprinting: insights from plants. *Annu. Rev. Genet.* 47 187–208 10.1146/annurev-genet-110711-155527 24016190

[B26] Gehring M. Bubb K. L. Henikoff S. (2009). Extensive demethylation of repetitive elements during seed development underlies gene imprinting. *Science* 324 1447–1451 10.1126/science.1171609 19520961 PMC2886585

[B27] Grant-Downton R. Kourmpetli S. Hafidh S. Khatab H. Le Trionnaire G. Dickinson H. (2013). Artificial microRNAs reveal cell-specific differences in small RNA activity in pollen. *Curr. Biol.* 23 R599–R601 10.1016/j.cub.2013.05.055 23885870

[B28] Greer E. Martin A. C. Pendle A. Colas I. Jones A. M. Moore G. (2012). The Ph1 locus suppresses Cdk2-type activity during premeiosis and meiosis in wheat. *Plant Cell* 24 152–162 10.1105/tpc.111.094771 22274628 PMC3289575

[B29] Grob S. Schmid M. W. Luedtke N. W. Wicker T. Grossniklaus U. (2013). Characterization of chromosomal architecture in *Arabidopsis* by chromosome conformation capture. *Genome Res.* 14:R129 10.1186/gb-2013-14-11-r129 PMC4053840 24267747

[B30] Gutierrez-Marcos J. F. Costa L. M. Dal Pra M. Scholten S. Kranz E. Perez P. (2006). Epigenetic asymmetry of imprinted genes in plant gametes. *Nat. Genet.* 38 876–878 10.1038/ng1828 16823380

[B31] Hackett J. A. Zylicz J. J. Surani M. A. (2012). Parallel mechanisms of epigenetic reprogramming in the germline. *Trends Genet.* 28 164–174 10.1016/j.tig.2012.01.005 22386917

[B32] Hajkova P. (2011). Epigenetic reprogramming in the germline: towards the ground state of the epigenome. *Philos. Trans. R. Soc. Lond. B Biol. Sci.* 366 2266–2273 10.1098/rstb.2011.0042 21727132 PMC3130423

[B33] Hajkova P. Ancelin K. Waldmann T. Lacoste N. Lange U. C. Cesari F. (2008). Chromatin dynamics during epigenetic reprogramming in the mouse germ line. *Nature* 452 877–881 10.1038/nature06714 18354397 PMC3847605

[B34] Hajkova P. Erhardtb S. Lanec N. Haafd T. El-Maarrie O. Reikc W. (2002). Epigenetic reprogramming in mouse primordial germ cells. *Mech. Dev.* 117 15–23 10.1016/S0925-4773(02)00181-8 12204247

[B35] Hoffmann R. D. Palmgren M. G. (2013). Epigenetic repression of male gametophyte-specific genes in the *Arabidopsis* sporophyte. *Mol. Plant* 6 1176–1186 10.1093/mp/sst100 23770838

[B36] Houben A. Kumke K. Nagaki K. Hause G. (2011). CENH3 distribution and differential chromatin modifications during pollen development in rye (*Secale cereale* L.). *Chromosome Res.* 19 471–480 10.1007/s10577-011-9207-6 21503764

[B37] Hsieh T. F. Ibarra C. A. Silva P. Zemach A. Eshed-Williams L. Fischer R. L. (2009). Genome-wide demethylation of *Arabidopsis* endosperm. *Science* 324 1451–1454 10.1126/science.1172417 19520962 PMC4044190

[B38] Ibarra C. A. Feng X. Schoft V. K. Hsieh T. F. Uzawa R. Rodrigues J. A. (2012). Active DNA demethylation in plant companion cells reinforces transposon methylation in gametes. *Science* 337 1360–1364 10.1126/science.1224839 22984074 PMC4034762

[B39] Ingouff M. Hamamura Y. Gourgues M. Higashiyama T. Berger F. (2007). Distinct dynamics of HISTONE3 variants between the two fertilization products in plants. *Curr. Biol.* 17 1032–1037 10.1016/j.cub.2007.05.019 17555967

[B40] Ingouff M. Rademacher S. Holec S. Soljic L. Xin N. Readshaw A. (2010). Zygotic resetting of the HISTONE 3 variant repertoire participates in epigenetic reprogramming in *Arabidopsis*. *Curr. Biol.* 20 2137–2143 10.1016/j.cub.2010.11.012 21093266

[B41] Ito H. Gaubert H. Bucher E. Mirouze M. Vaillant I. Paszkowski J. (2011). An siRNA pathway prevents transgenerational retrotransposition in plants subjected to stress. *Nature* 472 115–119 10.1038/nature09861 21399627

[B42] Jahnke S. Scholten S. (2009). Epigenetic resetting of a gene imprinted in plant embryos. *Curr. Biol.* 19 1677–1681 10.1016/j.cub.2009.08.053 19781944

[B43] Jenuwein T. Allis C. D. (2001). Translating the histone code. *Science* 293 1074–1080 10.1126/science.1063127 11498575

[B44] Jullien P. E. Berger F. (2010). DNA methylation reprogramming during plant sexual reproduction? *Trends Genet.* 26 394–399 10.1016/j.tig.2010.06.001 20609490

[B45] Jullien P. E. Mosquna A. Ingouff M. Sakata T. Ohad N. Berger F. (2008). Retinoblastoma and its binding partner MSI1 control imprinting in *Arabidopsis*. *PLoS Biol.* 6:e194 10.1371/journal.pbio.0060194 PMC2504488 18700816

[B46] Jullien P. E. Susaki D. Yelagandula R. Higashiyama T. Berger F. (2012). DNA methylation dynamics during sexual reproduction in *Arabidopsis thaliana*. *Curr. Biol.* 22 1825–1830 10.1016/j.cub.2012.07.061 22940470

[B47] Kniewel R. Keeney S. (2009). Histone methylation sets the stage for meiotic DNA breaks. *EMBO J.* 28 81–83 10.1038/emboj.2008.277 19158660 PMC2634739

[B48] Köhler C. Wolff P. Spillane C. (2012). Epigenetic mechanisms underlying genomic imprinting in plants. *Annu. Rev. Plant Biol.* 63 331–352 10.1146/annurev-arplant-042811-105514 22404470

[B49] Kumar S. V. Wigge P. A. (2010). H2A.Z-containing nucleosomes mediate the thermosensory response in *Arabidopsis. Cell* 140 136–147 10.1016/j.cell.2009.11.006 20079334

[B50] Law J. A. Jacobsen S. E. (2010). Establishing, maintaining and modifying DNA methylation patterns in plants and animals. *Nat. Rev. Genet.* 11 204–220 10.1038/nrg2719 20142834 PMC3034103

[B51] Lu F. Cui X. Zhang S. Jenuwein T. Cao X. (2011). *Arabidopsis* REF6 is a histone H3 lysine 27 demethylase. *Nat. Genet.* 43 715–719 10.1038/ng.854 21642989

[B52] Maheshwari P. (1950). *An Introduction to the Embryology of Angiosperms.* New York: McGraw-Hill

[B53] Mansour A. A. Gafni O. Weinberger L. Zviran A. Ayyash M. Rais Y. (2012). The H3K27 demethylase Utx regulates somatic and germ cell epigenetic reprogramming. *Nature* 488 409–413 10.1038/nature11272 22801502

[B54] Martinho R. G. Kunwar P. S. Casanova J. Lehmann R. (2004). A noncoding RNA is required for the repression of RNApolII-dependent transcription in primordial germ cells. *Curr. Biol.* 14 159–165 10.1016/j.cub.2003.12.036 14738740

[B55] Melamed-Bessudo C. Levy A. A. (2012). Deficiency in DNA methylation increases meiotic crossover rates in euchromatic but not in heterochromatic regions in *Arabidopsis*. *Proc. Natl. Acad. Sci. U.S.A.* 109 E981–E988 10.1073/pnas.1120742109 22460791 PMC3341010

[B56] Mirouze M. Lieberman-Lazarovich M. Aversano R. Bucher E. Nicolet J. Reinders J. (2012). Loss of DNA methylation affects the recombination landscape in *Arabidopsis*. *Proc. Natl. Acad. Sci. U.S.A.* 109 5880–5889 10.1073/pnas.1120841109 22451936 PMC3326504

[B57] Morales-Ruiz T. Ortega-Galisteo A. P. Ponferrada-Marin M. I. Martinez-Macias M. I. Ariza R. R. Roldan-Arjona T. (2006). DEMETER and REPRESSOR OF SILENCING 1 encode 5-methylcytosine DNA glycosylases. *Proc. Natl. Acad. Sci. U.S.A.* 103 6853–6858 10.1073/pnas.0601109103 16624880 PMC1458983

[B58] Mosher R. A. Melnyk C. W. Kelly K. A. Dunn R. M. Studholme D. J. Baulcombe D. C. (2009). Uniparental expression of PolIV-dependent siRNAs in developing endosperm of *Arabidopsis*. *Nature* 460 283–286 10.1038/nature08084 19494814

[B59] Nakamura A. Shirae-Kurabayashi M. Hanyu-Nakamura K. (2010). Repression of early zygotic transcription in the germline. *Curr. Opin. Cell Biol.* 22 709–714 10.1016/j.ceb.2010.08.012 20817425

[B60] Nonomura K. Morohoshi A. Nakano M. Eiguchi M. Miyao A. Hirochika H. (2007). A germ cell specific gene of the ARGONAUTE family is essential for the progression of premeiotic mitosis and meiosis during sporogenesis in rice. *Plant Cell* 19 2583–2594 10.1105/tpc.107.053199 17675402 PMC2002623

[B61] Ohno R. Nakayama M. Naruse C. Okashita N. Takano O. Tachibana M. (2013). A replication-dependent passive mechanism modulates DNA demethylation in mouse primordial germ cells. *Development* 140 2892–2903 10.1242/dev.093229 23760957

[B62] Olmedo-Monfil V. Durán-Figueroa N. Arteaga-Vázquez M. Demesa-Arévalo E. Autran D. Grimanelli D. (2010). Control of female gamete formation by a small RNA pathway in *Arabidopsis*. *Nature* 464 628–632 10.1038/nature08828 20208518 PMC4613780

[B63] Pecinka A. Schubert V. Meister A. Kreth G. Klatte M. Lysak M. A. (2004). Chromosome territory arrangement and homologous pairing in nuclei of *Arabidopsis thaliana* are predominantly random except for NOR-bearing chromosomes. *Chromosoma* 113 258–269 10.1007/s00412-004-0316-2 15480725

[B64] Pillot M. Baroux C. Vazquez M. A. Autran D. Leblanc O. Vielle-Calzada J. P. (2010). Embryo and endosperm inherit distinct chromatin and transcriptional states from the female gametes in *Arabidopsis*. *Plant Cell* 22 307–320 10.1105/tpc.109.071647 20139161 PMC2845419

[B65] Probst A. V. Fransz P. F. Paszkowski J. Scheid O. M. (2003). Two means of transcriptional reactivation within heterochromatin. *Plant J.* 33 743–749 10.1046/j.1365-313X.2003.01667.x 12609046

[B66] Qin Y. Zhao L. Skaggs M. I. Andreuzza S. Tsukamoto T. Panoli A. (2014). ACTIN-RELATED PROTEIN6 regulates female meiosis by modulating meiotic gene expression in *Arabidopsis*. *Plant Cell* 26 1612–1628 10.1105/tpc.113.120576 24737671 PMC4036575

[B67] Rabiger D. S. Drews G. N. (2013). MYB64 and MYB119 are required for cellularization and differentiation during female gametogenesis in *Arabidopsis thaliana*. *PLoS Genet.* 9:e1003783 10.1371/journal.pgen.1003783 PMC3778002 24068955

[B68] Raissig M. T. Baroux C. Grossniklaus U. (2011). Regulation and flexibility of genomic imprinting during seed development. *Plant Cell* 23 16–26 10.1105/tpc.110.081018 21278124 PMC3051244

[B69] Raissig M. T. Bemer M. Baroux C. Grossniklaus U. (2013a). Genomic imprinting in the *Arabidopsis* embryo is partly regulated by PRC2. *PLoS Genet.* 9:e1003862 10.1371/journal.pgen.1003862 PMC3854695 24339783

[B70] Raissig M. T. Gagliardini V. Jaenisch J. Grossniklaus U. Baroux C. (2013b). Efficient and rapid isolation of early-stage embryos from *Arabidopsis thaliana* seeds. *J. Vis. Exp.* 7:76 10.3791/50371 PMC3735039 23770918

[B71] Ravi M. Shibata F. Ramahi J. S. Nagaki K. Chen C. Murata M. (2011). Meiosis-specific loading of the centromere-specific histone CENH3 in *Arabidopsis thaliana*. *PLoS Genet.* 7:e1002121 10.1371/journal.pgen.1002121 PMC3111537 21695238

[B72] Rea M. Zheng W. Chen M. Braud C. Bhangu D. Rognan T. N. (2012). Histone H1 affects gene imprinting and DNA methylation in *Arabidopsis*. *Plant J.* 71 776–786 10.1111/j.1365-313X.2012.05028.x 22519754 PMC3429642

[B73] Reinders J. Mirouze M. Nicolet J. Paszkowski J. (2013). Parent-of-origin control of transgenerational retrotransposon proliferation in *Arabidopsis*. *EMBO Rep.* 14 823–828 10.1038/embor.2013.95 23835507 PMC3790068

[B74] Roudier F. Ahmed I. Berard C. Sarazin A. Mary-Huard T. Cortijo S. (2011). Integrative epigenomic mapping defines four main chromatin states in *Arabidopsis*. *EMBO J.* 30 1928–1938 10.1038/emboj.2011.103 21487388 PMC3098477

[B75] Russell S. D. Gou X. Wong C. E. Wang X. Yuan T. Wei X. (2012). Genomic profiling of rice sperm cell transcripts reveals conserved and distinct elements in the flowering plant male germ lineage. *New Phytol.* 195 560–573 10.1111/j.1469-8137.2012.04199.x 22716952

[B76] Sasaki Y. Yasuda H. Ohba Y. Harada H. (1990). Isolation and characterization of a novel nuclear protein from pollen mother cells of lily. *Plant Physiol.* 94 1467–1471 10.1104/pp.94.3.1467 16667855 PMC1077400

[B77] Schmidt A. Schmid M. W. Grossniklaus U. (2012). Analysis of plant germline development by high-throughput RNA profiling: technical advances and new insights. *Plant J.* 70 18–29 10.1111/j.1365-313X.2012.04897.x 22449040

[B78] Schmidt A. Wuest S. E. Vijverberg K. Baroux C. Kleen D. Grossniklaus U. (2011). Transcriptome analysis of the *Arabidopsis* megaspore mother cell uncovers the importance of RNA helicases for plant germline development. *PLoS Biol.* 9:e1001155 10.1371/journal.pbio.1001155 PMC3176755 21949639

[B79] Schoft V. K. Chumak N. Mosiolek M. Slusarz L. Komnenovic V. Brownfield L. (2009). Induction of RNA-directed DNA methylation upon decondensation of constitutive heterochromatin. *EMBO Rep.* 10 1015–1021 10.1038/embor.2009.152 19680290 PMC2750062

[B80] Schubert V. Lermontova I. Schubert I. (2014). Loading of the centromeric histone H3 variant during meiosis-how does it differ from mitosis? *Chromosoma* 10.1007/s00412-014-0466-9 [Epub ahead of print] 24806806

[B81] Schulz P. Jensen W. A. (1981). Pre-fertilization in *Capsella*: ultrastructure and ultrachemical localization of acid phosphatase in female meiocytes. *Protoplasma* 107 27–45 10.1007/BF01275605

[B82] Seisenberger S. Peat J. R. Hore T. A. Santos F. Dean W. Reik W. (2013). Reprogramming DNA methylation in the mammalian life cycle: building and breaking epigenetic barriers. *Philos. Trans. R. Soc. Lond. B Biol. Sci.* 368:20110330 10.1098/rstb.2011.0330 PMC3539359 23166394

[B83] Seydoux G. Braun R. E. (2006). Pathway to totipotency: lessons from germ cells. *Cell* 127 891–904 10.1016/j.cell.2006.11.016 17129777

[B84] She W. Grimanelli D. Baroux C. (2014). An efficient method for quantitative, single-cell analysis of chromatin modification and nuclear architecture in whole-mount ovules in *Arabidopsis*. *J. Vis. Exp.* 88 e51530. 10.3791/51530 PMC4195603 24998753

[B85] She W. Grimanelli D. Rutowicz K. Whitehead M. W. Puzio M. Kotlinski M. (2013). Chromatin reprogramming during the somatic-to-reproductive cell fate transition in plants. *Development* 140 4008–4019 10.1242/dev.095034 24004947

[B86] Singh M. Goel S. Meeley R. B. Dantec C. Parrinello H. Michaud C. (2011). Production of viable gametes without meiosis in maize deficient for an ARGONAUTE protein. *Plant Cell* 23 443–458 10.1105/tpc.110.079020 21325139 PMC3077773

[B87] Slotkin R. K. Vaughn M. Borges F. Tanurdzic M. Becker J. D. Feijo J. A. (2009). Epigenetic reprogramming and small RNA silencing of transposable elements in pollen. *Cell* 136 461–472 10.1016/j.cell.2008.12.038 19203581 PMC2661848

[B88] Sniezko R. (2006). “Meiosis in plants,” in *Plant Cell Biology,* eds Dashek W. V. Harrison P. (New Hampshire: Science Publisher) 227–258

[B89] Sprunck S. Gross-Hardt R. (2011). Nuclear behavior, cell polarity, and cell specification in the female gametophyte. *Sex. Plant Reprod.* 24 123–136 10.1007/s00497-011-0161-4 21336612

[B90] Stroud H. Otero S. Desvoyes B. Ramírez-Parra E. Jacobsen S. E. Gutierrez C. (2012). Genome-wide analysis of histone H3.1 and H3.3 variants in *Arabidopsis thaliana*. *Proc. Natl. Acad. Sci. U.S.A.* 10.1073/pnas.1203145109 PMC3325649 22431625

[B91] Surani M. A. Hayashi K. Hajkova P. (2007). Genetic and epigenetic regulators of pluripotency. *Cell* 128 747–762 10.1016/j.cell.2007.02.010 17320511

[B92] Tachibana M. Nozaki M. Takeda N. Shinkai Y. (2007). Functional dynamics of H3K9 methylation during meiotic prophase progression. *EMBO J.* 26 3346–3359 10.1038/sj.emboj.7601767 17599069 PMC1933398

[B93] Tanaka I. Ono K. Fukuda T. (1998). The developmental fate of angiosperm pollen is associated with a preferential decrease in the level of histone H1 in the vegetative nucleus. *Planta* 206 561–569 10.1007/s004250050433

[B94] Tariq M. Paszkowski J. (2004). DNA and histone methylation in plants. *Trends Genet.* 20 244–251 10.1016/j.tig.2004.04.005 15145577

[B95] Ueda K. Kinoshita Y. Xu Z. Ide N. Ono M. Akahori Y. (2000). Unusual core histones specifically expressed in male gametic cells of *Lilium longiflorum*. *Chromosoma* 108 491–500 10.1007/s004120050401 10794571

[B96] van Zanten M. Koini M. A. Geyer R. Liu Y. Brambilla V. Bartels D. (2011). Seed maturation in *Arabidopsis thaliana* is characterized by nuclear size reduction and increased chromatin condensation. *Proc. Natl. Acad. Sci. U.S.A.* 108 20219–20224 10.1073/pnas.1117726108 22123962 PMC3250172

[B97] Vaucheret H. (2008). Plant ARGONAUTES. *Trends Plant Sci.* 13 350–358 10.1016/j.tplants.2008.04.007 18508405

[B98] Wendrich J. R. Weijers D. (2013). The *Arabidopsis* embryo as a miniature morphogenesis model. *New Phytol.* 199 14–25 10.1111/nph.12267 23590679

[B99] Wierzbicki A. T. Jerzmanowski A. (2005). Suppression of histone H1 genes in *Arabidopsis* results in heritable developmental defects and stochastic changes in DNA methylation. *Genetics* 169 997–1008 10.1534/genetics.104.031997 15489532 PMC1449090

[B100] Wohrmann H. J. Gagliardini V. Raissig M. T. Wehrle W. Arand J. Schmidt A. (2012). Identification of a DNA methylation-independent imprinting control region at the *Arabidopsis* MEDEA locus. *Genes Dev.* 26 1837–1850 10.1101/gad.195123.112 22855791 PMC3426762

[B101] Wuest S. E. Schmid M. W. Grossniklaus U. (2013). Cell-specific expression profiling of rare cell types as exemplified by its impact on our understanding of female gametophyte development. *Curr. Opin. Plant Biol.* 16 41–49 10.1016/j.pbi.2012.12.001 23276786

[B102] Wuest S. E. Vijverberg K. Schmidt A. Weiss M. Gheyselinck J. Lohr M. (2010). *Arabidopsis* female gametophyte gene expression map reveals similarities between plant and animal gametes. *Curr. Biol.* 20 506–512 10.1016/j.cub.2010.01.051 20226671

[B103] Xu C. Tian J. Mo B. (2013). siRNA-mediated DNA methylation and H3K9 dimethylation in plants. *Protein Cell* 10.1007/s13238-013-3052-7 [Epub ahead of print] PMC4875530 23943321

[B104] Xu H. Swoboda I. Bhalla P. Singh M. B. (1999). Male gametic cell-specific expression of H2A and H3 histone genes. *Plant Mol. Biol.* 39 607–614 10.1023/A:1006162120037 10092186

[B105] Yamaji M. Seki Y. Kurimoto K. Yabuta Y. Yuasa M. Shigeta M. (2008). Critical function of Prdm14 for the establishment of the germ cell lineage in mice. *Nat. Genet.* 40 1016–1022 10.1038/ng.186 18622394

[B106] Yang H. Lu P. Wang Y. Ma H. (2011). The transcriptome landscape of *Arabidopsis* male meiocytes from high-throughput sequencing: the complexity and evolution of the meiotic process. *Plant J.* 65 503–516 10.1111/j.1365-313X.2010.04439.x 21208307

[B107] Yoshida S. Barbier De Reuille P. Lane B. Bassel G. W. Prusinkiewicz P. Smith R. S. (2014). Genetic control of plant development by overriding a geometric division rule. *Dev. Cell* 29 75–87 10.1016/j.devcel.2014.02.002 24684831

[B108] Zemach A. Kim M. Y. Hsieh P.-H. Coleman-Derr D. Eshed-Williams L. Thao K. (2013). The *Arabidopsis* nucleosome remodeler DDM1 allows DNA methyltransferases to access H1-containing heterochromatin. *Cell* 153 193–205 10.1016/j.cell.2013.02.033 23540698 PMC4035305

[B109] Zemach A. Kim M. Y. Silva P. Rodrigues J. A. Dotson B. Brooks M. D. (2010). Local DNA hypomethylation activates genes in rice endosperm. *Proc. Natl. Acad. Sci. U.S.A.* 107 18729–18734 10.1073/pnas.1009695107 20937895 PMC2972920

[B110] Zhang D. Luo X. Zhu L. (2011). Cytological analysis and genetic control of rice anther development. *J. Genet. Genomics* 38 379–390 10.1016/j.jgg.2011.08.001 21930097

